# Supplementary material for: Clonal expansion and diversification of germinal center and memory B cell responses to booster immunization in primates
Source: Cell Rep. Author manuscript; Available in PMC 2025 Nov 14. (PMC12617206; doi:10.1016/j.celrep.2025.116142)
Supplement: 1 [file NIHMS2107294-supplement-1.pdf]

Cell Reports, Volume *44*

Supplemental information

Clonal expansion and diversification of germinal center and memory B cell responses to booster immunization in primates

Lachlan P. Deimel, Yoshiaki Nishimura, Gabriela S. Silva Santos, Viren A. Baharani, Brianna Hernandez, Thiago Y. Oliveira, Andrew J. MacLean, Marie Canis, Sadman Shawraz, Anna Gazumyan, Harald Hartweger, Paul D. Bieniasz, Theodora Hatziioannou, Malcolm A. Martin, and Michel C. Nussenzweig

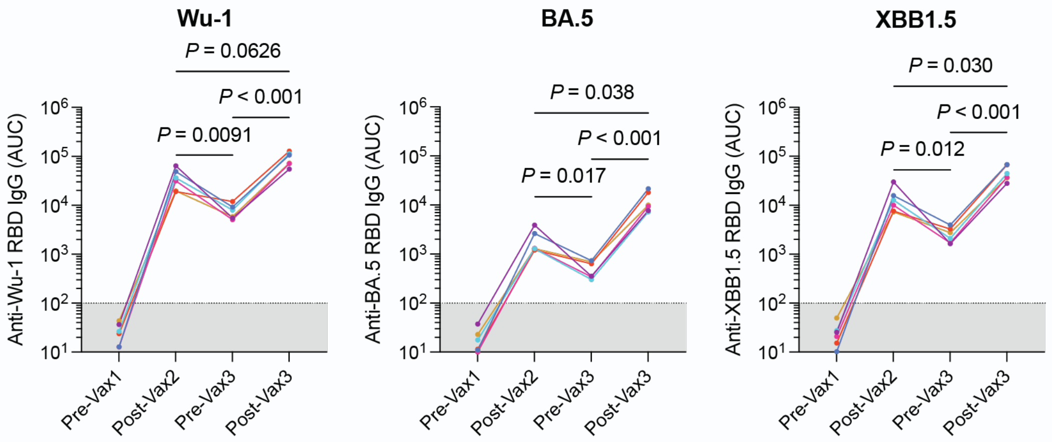

Figure S1: Plasma reactivity against SARS-CoV-2 RBD variants following immunization.

Plots showing IgG reactivity of plasma from rhesus macaques iteratively immunized with Pfizer Comirnaty 2023–24. Plasma was tested against RBD variants. Connected dots indicate data from the same animal at different timepoints. Comparisons were conducted using paired Tukey's post-hoc comparisons.

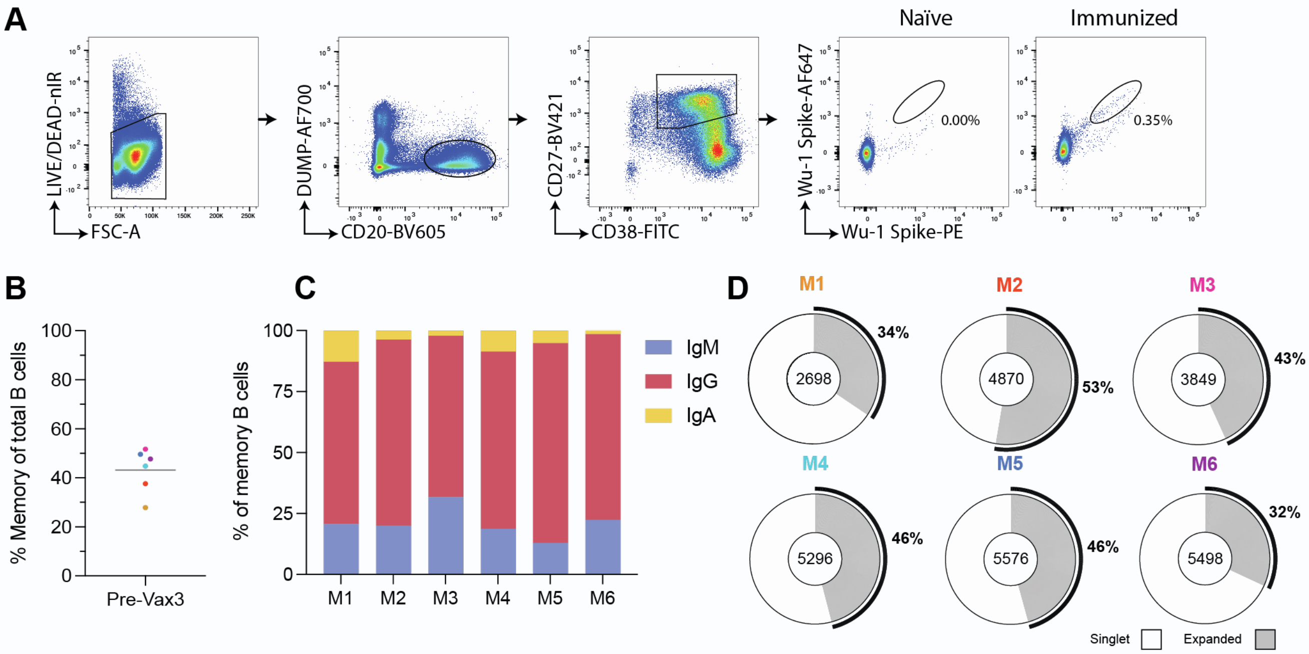

Figure S2: Circulating memory B cell clonality in rhesus macaques.

(A) Flow cytometry plots showing the gating used to select memory B cells (CD38⁺CD27⁺) in PBMCs and representative bait-binding profile in macaques.

(B) Plot showing the percentage of circulating memory B cells pre-vax3 in each animal. Each dot is the value from a single animal, colorized according to the macaque ID. Bar denotes the mean.

(C and D) Bulk memory B cell antibody sequences were acquired using the 10X genomics platform. **(C)** Graph shows the distribution of isotypes represented in the memory compartment for each macaque. **(D)** Donut plots showing the distribution of expanded versus singlet memory clones. The number of heavy and light chain variable region pairs obtained from each animal is shown in the middle of the donut. Grey segment and marked percentages reflect the proportion of cells belonging to expanded clones, while white denotes singlets.

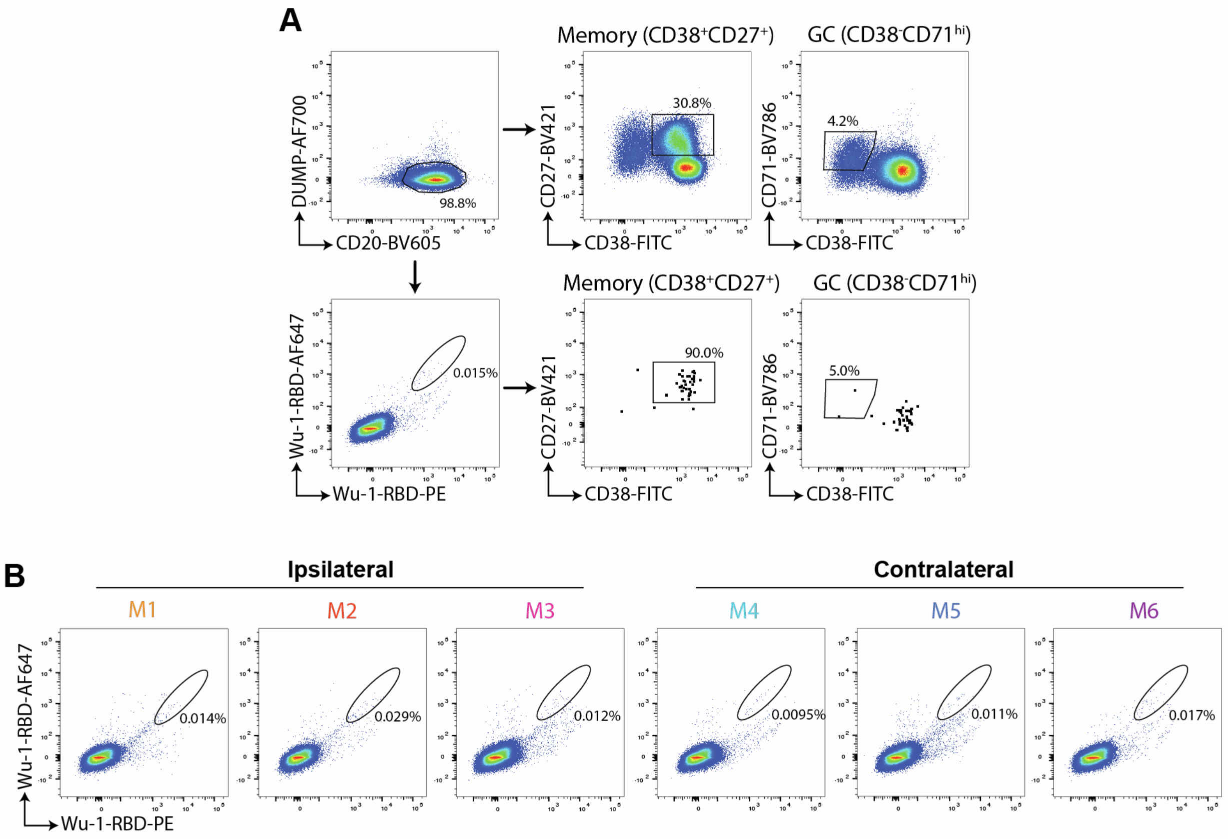

Figure S3: RBD-specific B cells in macaque spleen following booster immunizations.

(A) Flow cytometry plots of splenic macaque B cells. Plots show back-gating on Wu-1 bait-binding B cells.

(B) Plots showing the population of splenic memory B cells that bind RBD in each animal. Spleens were harvested at wk 15 following the third immunization.

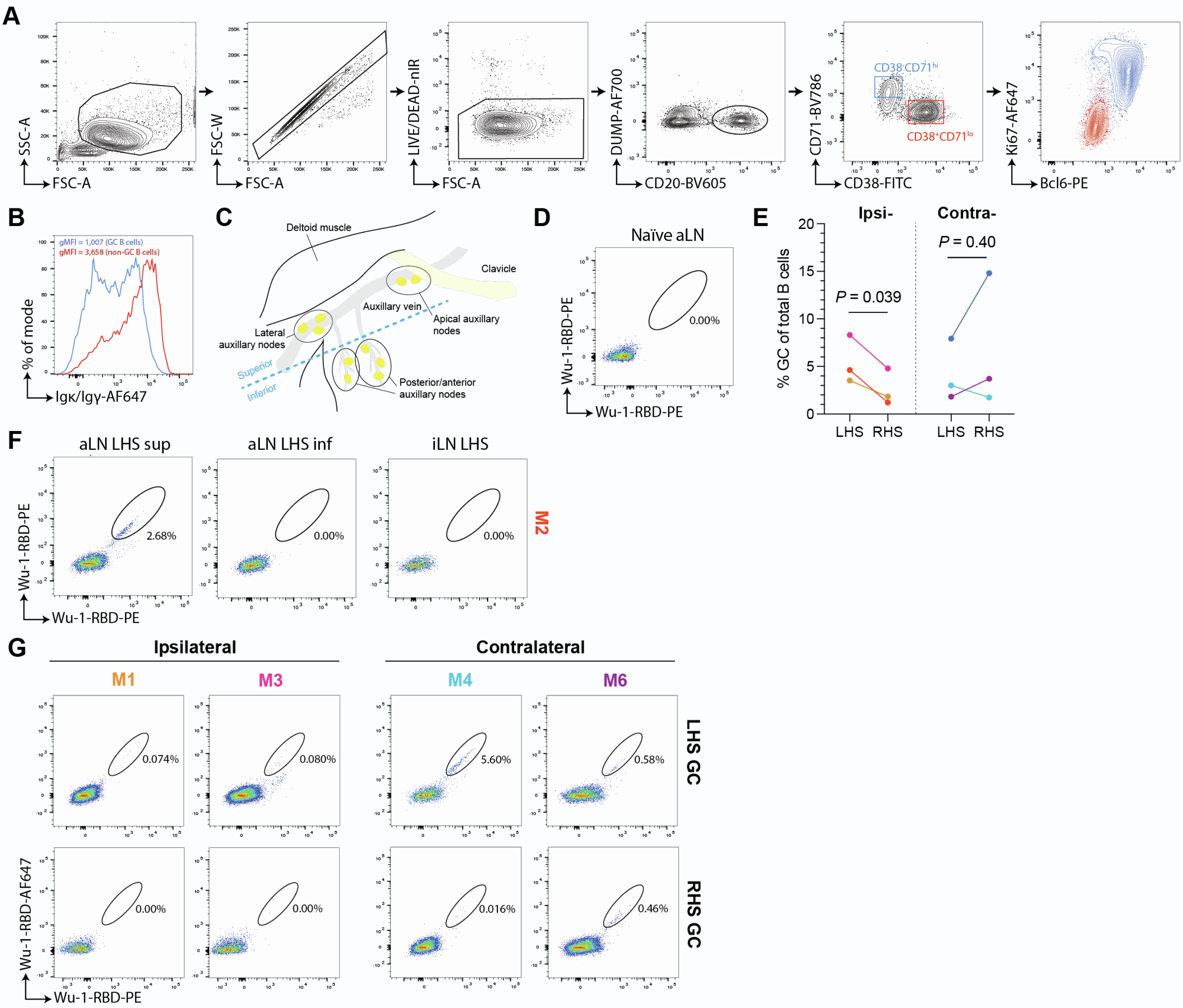

Figure S4: Flow cytometric analysis of booster responses in Rhesus macaques.

(A) Contour plots showing the lineage markers $CD20^+CD38^-CD71^{hi}$ that efficiently identifies GC B cells in the lymph nodes of Rhesus macaques.

(B) Histogram showing the distribution of surface BCR densities on GC and non-GC B cells.

(C) Schema highlighting the anatomy of major auxiliary node clusters in a Rhesus macaque.

(D) Flow cytometry plot of Wu-1 RBD bait-binding on aLN GC B cells from a naïve macaque.

(E) Plot showing the percentage of GC B cells in the respective superior aLN. Connected dots indicate data from the same animal. Data were compared using paired Student's t-tests.

(F) Representative pseudocolour plots showing RBD bait-binding on pre-gated GC B cells isolated from either the superior auxiliary lymph node (sup aLN), the inferior (inf) aLN or the inguinal (i)LNs.

(G) Flow cytometry plots showing RBD bait-binding on GC B cells in the post-boost sup aLN.

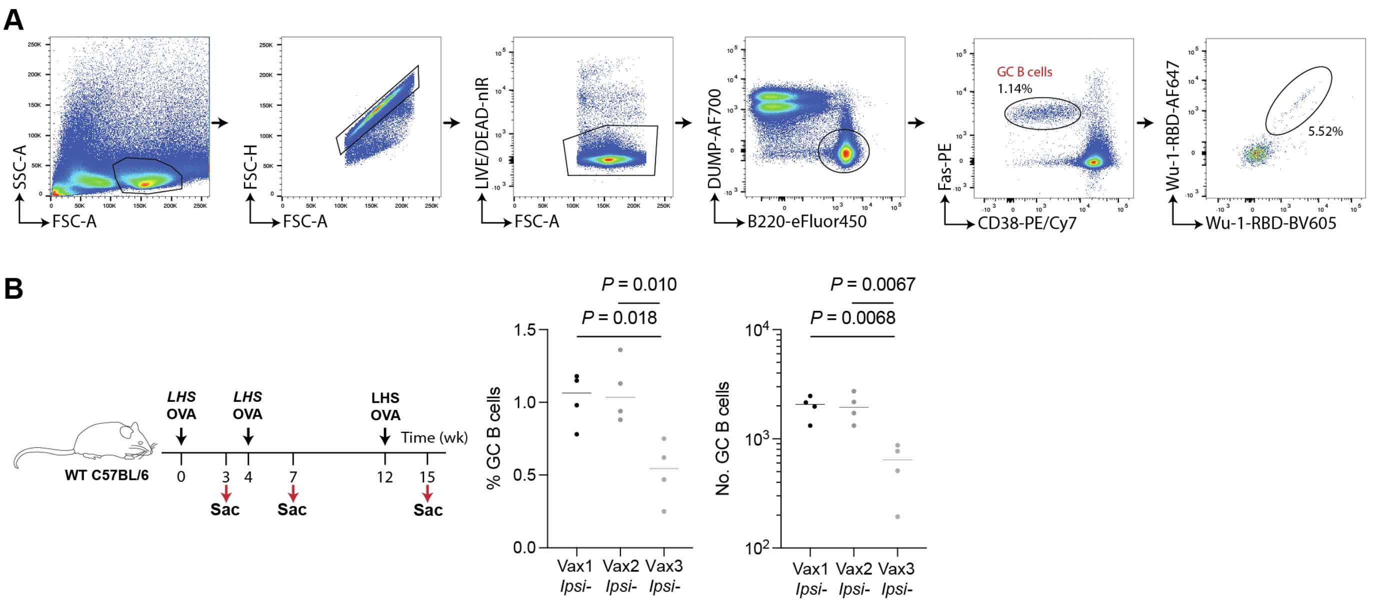

Figure S5: Germinal center B cell reactions following iterative immunizations in mice.

(A) Mouse GC and RBD-bait binding B cell gating strategy.

(B) Experimental scheme of immunization schedule (left) and evaluation of GC size after immunization (right).

Figure S6: Clone sharing and V_H gene distribution of post-boost RBD-binding B cells.

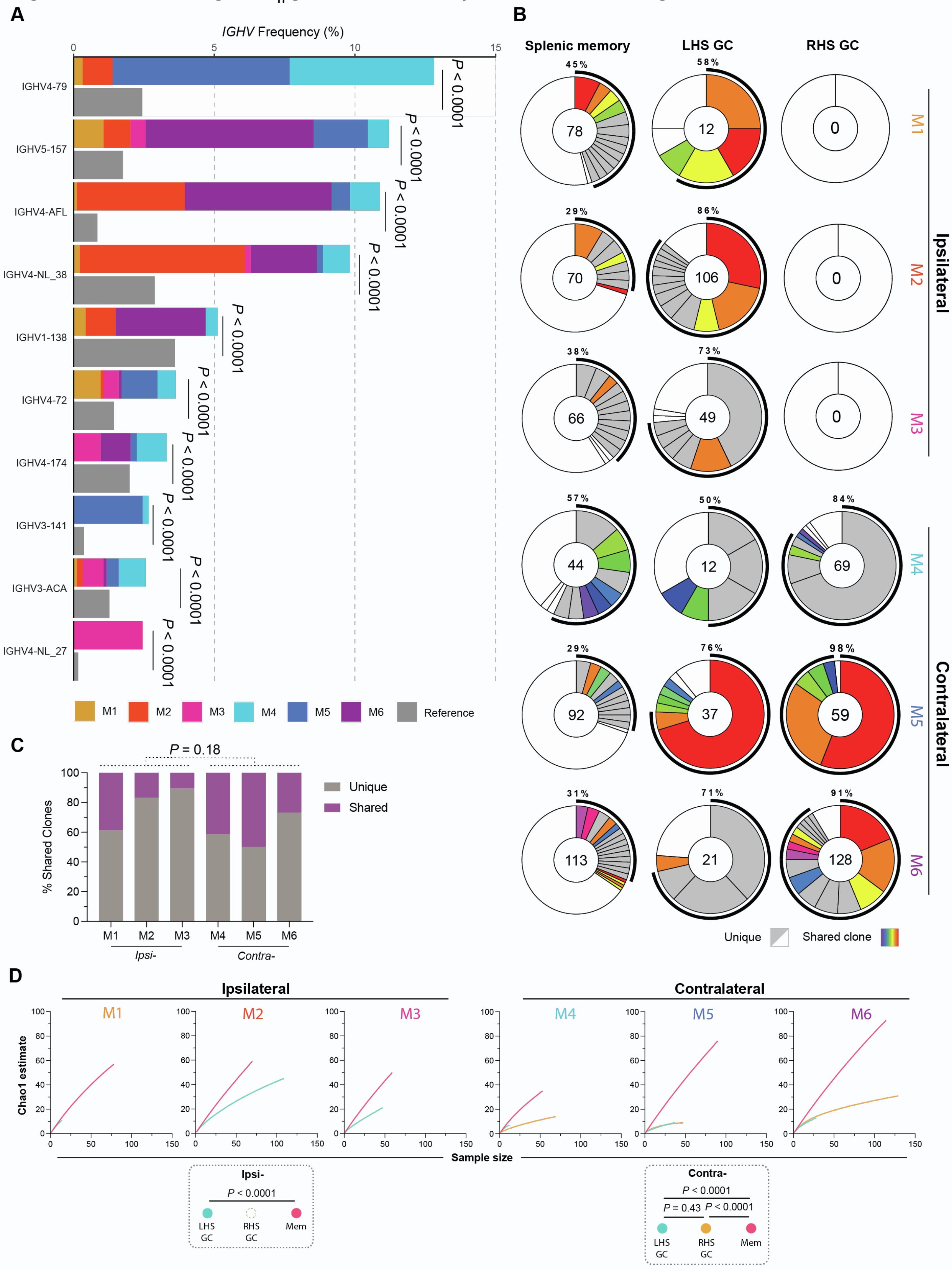

Figure S6: Clone sharing and V_H gene distribution of post-boost RBD-binding B cells.

(A) Bar plot shows the relative abundance of the 10 most common *IGHV* gene segments utilized by all RBD bait-binding B cells sequenced. Bars were partitioned and colorized according to animal identity. This was compared to the bulk circulating memory B cell library (fig. S2; and table S2). Statistical significance was measured using a two-sided binomial test with Benjamini-Hochberg *P*-value correction.

(B) Donut plots showing the clonality of Wu-1 RBD bait-binding B cells from both memory and GCs. The number of sequences reflected in each plot is shown in the center of each plot. Colored/grey segments denote expanded clones, with their size proportional to the number of clone members. The outer ring segment and marked percentage represents the proportion of cells belonging to an expanded clone. White segments denote singlets. Colored segments for each given animal correspond to clones present across compartments. ND = not detected/no sequences recovered.

(C) Plot showing the proportion of clonal families shared across multiple compartments or unique to a single compartment. Boost condition effects were tested using a Student's t test.

(D) Plots showing the Chao1 estimate traces for both memory and GC B cells. Compartment diversity was compared using a Kolmogorov-Smirnov test.

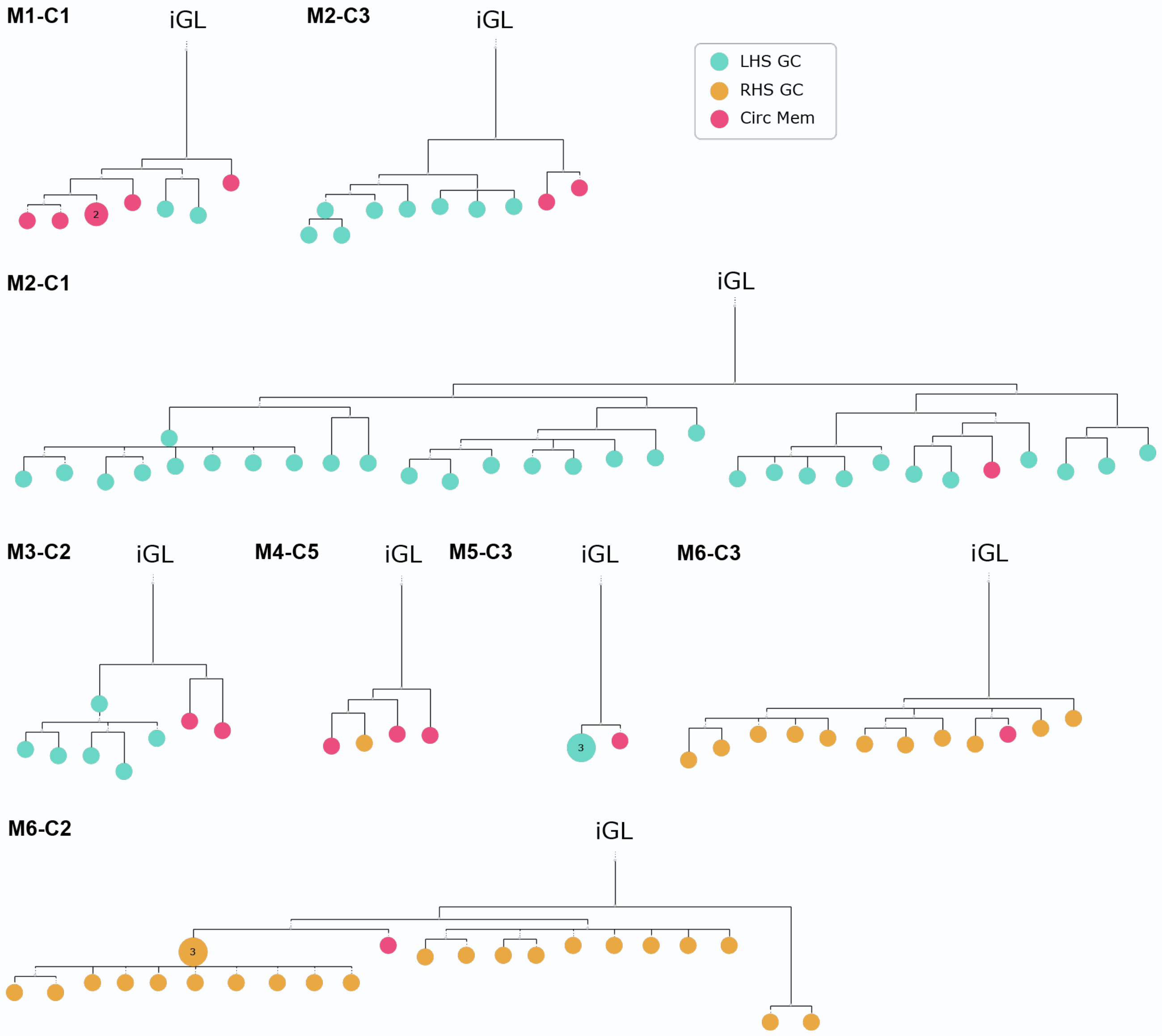

Figure S7: Representative trees of clones shared between the memory and GC compartments.

Phylogenetic trees of representative B cell clonal families shared between the GC and memory. Distance is proportional to the heavy and light chain sequence disparity, rooted to their inferred germline revertant (iGL). Unless otherwise marked, all nodes comprise of a single cell. Node color denotes the location and fate of the originating cell.

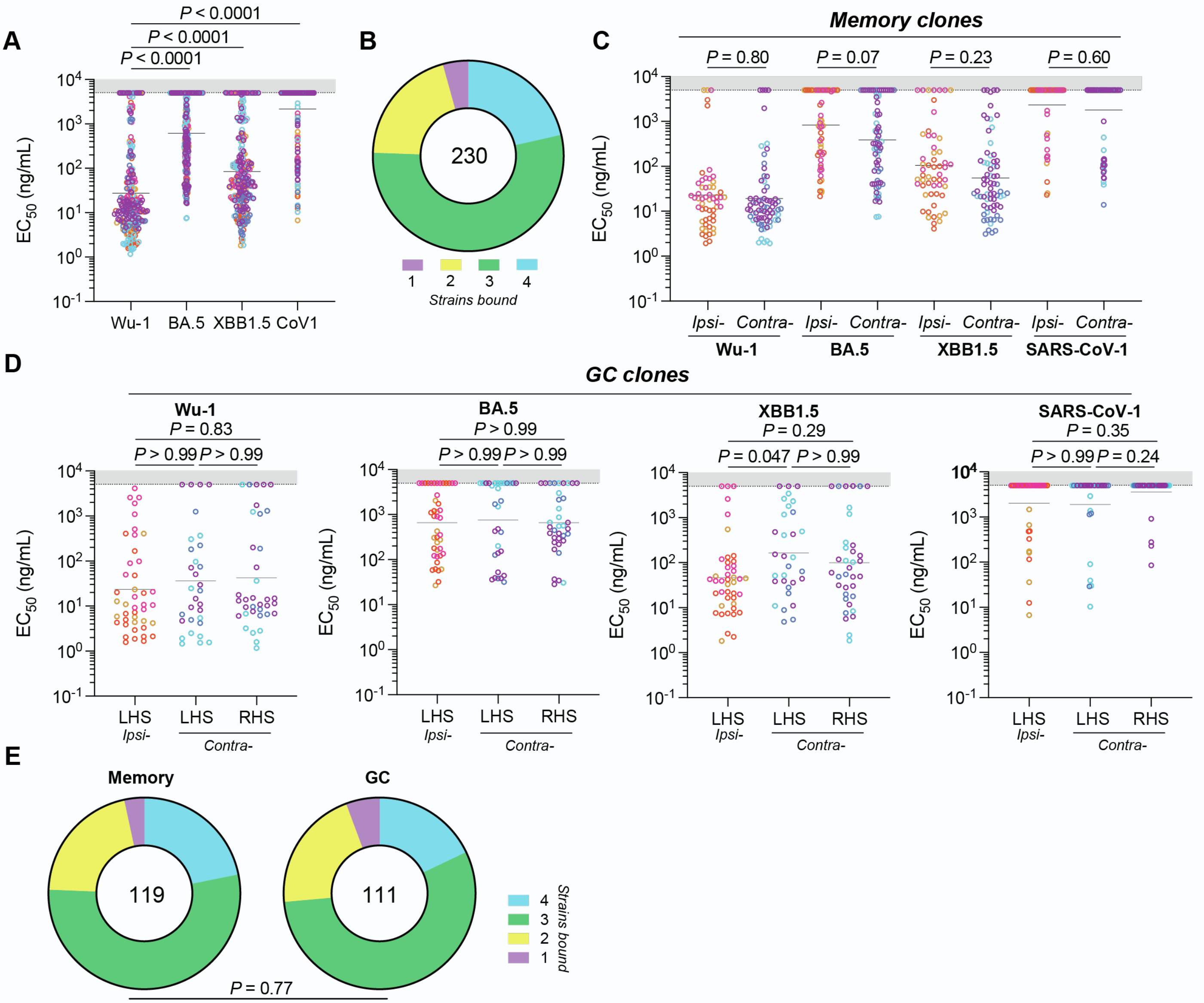

Figure S8: Binding and breadth of anti-RBD monoclonal antibodies.

(A) Plots showing the EC_{50} values of monoclonal antibodies cloned from memory and GC B cells screened against RBD variants via ELISA. The total number of monoclonal antibodies tested was 247.

(B) Donut plot showing the cross-variant RBD binding for antibodies reactive with Wu-1 RBD. Strains were deemed binders if EC_{50} < 5 µg/mL.

(C) Graph showing RBD variant binding of antibodies cloned from memory B cells in both ipsilaterally and contralaterally boosted animals. Vax3 site conditions were compared using Mann-Whitney tests.

(D) Plots show RBD binding of antibodies cloned from GC B cells.

(A and **D)** Groups were compared using Kruskal-Wallis tests.

(A, C and **D)** Dots show the EC_{50} value of a monoclonal antibody an indicated RBD variant. Dots are colorized according to the animal an antibody was cloned from. Bars denote the geometric mean.

(E) Donut plots showing the RBD reactivity breadth of antibodies derived from memory or GC B cells. Groups were compared using a chi-squared test.

(B and **E)** Clones where Wu-1 RBD EC_{50} ≥ 5 000 ng/mL was excluded from this analysis.

Figure S9: Variation in RBD reactivity of monoclonal antibodies via ELISA.

Group	Antibody	Wu-1	BA.5	XBB1.5	CoV1
Ipsi-	M1-C2-Mem-a	4	1021	23	5000
Ipsi-	M1-C2-LHS-a	13	1152	33	5000
Ipsi-	M1-C2-LHS-b	4	429	12	1479
Ipsi-	M1-C3-Mem-a	6	267	36	5000
Ipsi-	M1-C3-Mem-b	35	5000	524	5000
Ipsi-	M1-C3-LHS-b	6	240	50	174
Ipsi-	M1-C4-Mem-a	12	310	49	5000
Ipsi-	M1-C4-Mem-a	19	795	58	5000
Ipsi-	M1-C4-LHS-a	23	307	47	668
Ipsi-	M1-C10-Mem-a	4	26	7	25
Ipsi-	M1-C10-LHS-a	4	61	11	36
Ipsi-	M2-C2-Mem-a	3	201	10	5000
Ipsi-	M2-C2-Mem-b	5	5000	23	5000
Ipsi-	M2-C2-Mem-c	5	5000	7	5000
Ipsi-	M2-C2-LHS-b	167	1200	132	5000
Ipsi-	M2-C3-Mem-a	7	414	106	5000
Ipsi-	M2-C3-Mem-b	11	559	42	5000
Ipsi-	M2-C3-LHS-a	19	86	16	5000
Ipsi-	M2-C3-LHS-c	20	122	21	5000
Ipsi-	M3-C6-Mem-a	19	765	262	5000
Ipsi-	M3-C6-LHS-a	16	135	66	5000
Contra-	M4-C4-Mem-a	2	916	18	45
Contra-	M4-C4-Mem-b	2	4315	1125	99
Contra-	M4-C4-LHS-a	2	5000	132	10
Contra-	M4-C4-LHS-b	1	5000	53	39
Contra-	M4-C6-Mem-a	18	619	110	133
Contra-	M4-C6-Mem-b	6	357	122	39
Contra-	M4-C6-LHS-a	25	3825	2312	2928
Contra-	M4-C9-Mem-a	8	1470	174	5000
Contra-	M4-C9-Mem-b	6	246	62	5000
Contra-	M4-C9-RHS-a	7	2990	214	5000
Contra-	M5-C1-LHS-b	183	2005	64	5000
Contra-	M5-C1-LHS-c	224	1706	83	5000
Contra-	M5-C1-RHS-b	190	2303	49	5000
Contra-	M5-C1-RHS-c	1119	2152	55	5000
Contra-	M5-C2-Mem-b	61	590	22	5000
Contra-	M5-C2-LHS-b	7	406	28	1143
Contra-	M5-C2-RHS-b	10	375	18	5000
Contra-	M5-C2-RHS-c	6	450	16	5000
Contra-	M5-C6-Mem-a	5	33	6	5000
Contra-	M5-C6-Mem-b	5	35	6	5000
Contra-	M5-C6-LHS-a	4	38	9	1200
Contra-	M5-C8-Mem-a	35	225	25	5000
Contra-	M5-C8-Mem-8b	11	88	8	5000
Contra-	M5-C8-LHS-a	22	127	21	5000
Contra-	M5-C13-Mem-a	5	5000	6	14
Contra-	M5-C13-LHS-a	5	32	5	29
Contra-	M6-C1-Mem-a	60	188	52	5000
Contra-	M6-C1-Mem-b	90	107	43	5000
Contra-	M6-C1-LHS-a	71	140	140	5000
Contra-	M6-C1-RHS-a	200	241	49	5000
Contra-	M6-C1-RHS-b	5000	664	118	5000
Contra-	M6-C2-Mem-a	5000	5000	5000	5000
Contra-	M6-C2-RHS-a	11	28	12	5000
Contra-	M6-C2-RHS-b	10	219	28	5000
Contra-	M6-C3-Mem-a	7	5000	133	5000
Contra-	M6-C3-RHS-a	1738	5000	5000	5000
Contra-	M6-C3-RHS-b	14	531	109	5000
Contra-	M6-C4-Mem-a	9	121	40	73
Contra-	M6-C4-Mem-b	9	476	61	56
Contra-	M6-C4-RHS-a	13	263	48	279
Contra-	M6-C4-RHS-a	15	382	60	85
Contra-	M6-C10-Mem-a	1959	5000	1384	5000
Contra-	M6-C10-Mem-b	244	678	133	5000
Contra-	M6-C10-RHS-a	72	293	32	5000
Contra-	M6-C10-RHS-b	5000	5000	5000	5000
Contra-	M6-C13-Mem-a	48	315	201	146
Contra-	M6-C13-RHS-a	11	384	177	5000
Contra-	M6-C13-RHS-b	7	483	144	5000
Contra-	M6-C15-Mem-a	15	5000	87	5000
Contra-	M6-C15-RHS-a	6	34	6	5000
Contra-	M6-C15-RHS-b	8	37	6	5000

EC_{50} values in ng/mL

Figure S9: Variation in RBD reactivity of monoclonal antibodies via ELISA.

Heatmap showing the EC_{50} values of monoclonal antibody reactivities against RBD variants via ELISA. Cells are clustered by shared clones, and thus antibodies derived from cells present in multiple compartments were evaluated. mAb ID assignments correspond to the monkey (M*x*), clone number (C*y*), origin (memory, LHS/RHS GC) and cell identifier (a,b, etc.).

SI Table 1: Indicidual rhesus macaque characteristics

Animal Number	Animal IDs	Age	Sex
**M1**	DGZ8	7 years, 3 months	M
**M2**	DGK7	7 years, 4 months	M
**M3**	DGX6	7 years, 8 months	M
**M4**	DI0N	4 years, 5 months	M
**M5**	H920	7 years, 4 months	M
**M6**	DHBH	7 years, 8 months	M
